# Altered Plasma Lysophosphatidylcholines and Amides in Non-Obese and Non-Diabetic Subjects with Borderline-To-Moderate Hypertriglyceridemia: A Case-Control Study

**DOI:** 10.1371/journal.pone.0123306

**Published:** 2015-04-09

**Authors:** Sae Young Lee, Minjoo Kim, Saem Jung, Sang-Hyun Lee, Jong Ho Lee

**Affiliations:** 1 Interdisciplinary Course of Science for Aging, Yonsei University, Seoul, Korea; 2 Research Center for Silver Science, Institute of Symbiotic Life-TECH, Yonsei University, Seoul, Korea; 3 National Leading Research Laboratory of Clinical Nutrigenetics/Nutrigenomics, Department of Food and Nutrition, College of Human Ecology, Yonsei University, Seoul, Korea; 4 Department of Food and Nutrition, Brain Korea 21 PLUS Project, College of Human Ecology, Yonsei University, Seoul, Korea; 5 Department of Family Practice, National Health Insurance Corporation Ilsan Hospital, Goyang, Korea; 6 Cardiovascular Research Institute, Yonsei University College of Medicine, Seoul, Korea; Steno Diabetes Center, DENMARK

## Abstract

Hypertriglyceridemia (HTG) is a risk factor for atherosclerotic cardiovascular disease (CVD). We investigated alterations in plasma metabolites associated with borderline-to-moderate HTG (triglycerides (TG) 150-500 mg/dL). Using UPLC-LTQ-Orbitrap mass spectrometry analysis, the metabolomics profiles of 111 non-diabetic and non-obese individuals with borderline-to-moderate HTG were compared with those of 111 age- and sex-matched controls with normotriglyceridemia (NTG, TG <150 mg/dL). When compared to the NTG control group, the HTG group exhibited higher plasma levels of lysophosphatidylcholines (lysoPCs), including C14:0 (*q* = 0.001) and C16:0 (*q* = 1.8E-05), and several amides, including N-ethyldodecanamide (*q* = 2.9E-05), N-propyldodecanamide (*q* = 3.5E-05), palmitoleamide (*q* = 2.9E-06), and palmitic amide (*q* = 0.019). The metabolomic profiles of the HTG group also exhibited lower plasma levels of cis-4-octenedioic acid (*q*<1.0E-9) and docosanamide (*q* = 0.002) compared with those of the NTG controls. LysoPC 16:0 and palmitoleamide emerged as the primary metabolites able to discriminate the HTG group from the NTG group in a partial least-squares discriminant analysis and were positively associated with the fasting triglyceride levels. We identified alterations in lysoPCs, amides, and cis-4-octenedioic acid among non-diabetic and non-obese individuals with borderline-to-moderate HTG. These results provide novel insights into the metabolic alterations that occur in the early metabolic stages of HTG. This information may facilitate the design of early interventions to prevent disease progression.

## Introduction

Hypertriglyceridemia (HTG) is a polygenic and environmentally determined metabolic disease that affects the production and clearance of triglyceride (TG)-rich lipoproteins [1]. An elevated level of plasma TG is an independent risk factor for type 2 diabetes (T2D), metabolic syndrome, and atherosclerotic cardiovascular disease (CVD) [2, 3], with a prevalence of up to 30–50% in these populations. Many studies have focused on the loci of genetic susceptibility [1] of HTG and its co-morbidity with several metabolic diseases, including obesity, T2D, and CVD. Borderline-to-moderate HTG, which is defined by TG levels of 150–500 mg/dL, precedes severe HTG, T2D, and CVD. However, borderline-to-moderate HTG-induced perturbations in metabolism have not been characterized among non-diabetic and non-obese individuals. Studies are needed to determine the role of altered plasma metabolic profiles in individuals who only have borderline-to-moderate HTG and do not have diabetes or obesity. Metabolomics is a potential tool to help understand disease pathophysiology [4, 5] if applied in a systems biology approach. The main aim of this study was to identify metabolic changes associated with borderline-to-moderate HTG levels in non-obese and non-diabetic individuals to uncover new insights regarding early metabolic perturbations associated with elevated TG levels.

For this purpose, we applied a metabolomics approach using plasma samples of 111 non-diabetic and non-obese individuals who remained within the range of borderline-to-moderate HTG during repeated measurements over a 3-month period and compared these profiles with those of 111 age- and sex-matched control patients with normotriglyceridemia (NTG, TG <150 mg/dL).

## Materials and Methods

### Study subjects

Study subjects were recruited from the Health Service Center (HSC) during routine check-ups at the National Health Insurance Corporation (NHIC) Ilsan Hospital in Goyang, Korea between June 2013 and March 2014. HTG was defined according to the National Cholesterol Education Program—Adult Treatment Panel III [6]. Based on the data screened from the HSC, subjects who were non-obese and non-diabetic with borderline-to-moderate HTG were referred to the Departments of Family Medicine and Internal Medicine. This screening procedure identified 111 non-obese and non-diabetic individuals who maintained borderline-to-moderate HTG levels during repeated measurements over a 3-month period and 111 age- and sex-matched control subjects who maintained NTG levels during repeated measurements over a 3-month period. The purpose of the study was explained to all participants, and written consent was obtained prior to their participation. The Institutional Review Boards of the Ilsan Hospital and Yonsei University provided ethical approval of the study protocol, which was performed according to the Declaration of Helsinki. Subjects were excluded from the study if they had taken lipid-lowering medications, any medications or supplements known to affect lipid metabolism, or any probiotics products in the past month; had any diagnosis of dyslipidemia, diabetes mellitus, hypertension, liver disease, renal disease, CVD, cerebrovascular disease, pancreatitis, cancer, or medication or alcohol abuse; or were pregnant or breastfeeding.

### Clinical measurements

Body weight (UM0703581; Tanita, Tokyo, Japan) and height (GL-150; G-tech International, Uijeongbu, Korea) measurements of unclothed subjects without shoes were measured in the morning to calculate the BMI (kg/m^2^). Waist circumference was measured at the umbilical level after normal expiration with the subject in an upright standing posture, and hip girth was measured at the widest part of the hip using a plastic measuring tape with measurements to the nearest 0.1 cm. These measurements were obtained to calculate the waist-to-hip ratio (WHR). Blood pressure (BP) was measured in the left arm of seated patients with an automatic BP monitor (TM-2654; A&D, Tokyo, Japan) after 20 min of rest. Venous blood specimens were collected after a 12-hour fasting period in EDTA-treated and untreated tubes. The blood specimens collected in the EDTA-treated tubes were used for separation into plasma, and the specimens in the untreated tubes were separated into serum. Then, the specimens were aliquoted and stored at −80°C until further analysis. The time from blood sample collection to storage was less than 3 h. The usual dietary intake of the study subjects was assessed using a semi-quantitative food frequency questionnaire and a 24-hour recall method. The nutrient intake of each subject was determined and calculated based on 3 days of food records using the Computer-Aided Nutritional Analysis Program (CAN-pro 3.0; Korean Nutrition Society, Seoul, Korea).

Fasting levels of total cholesterol and TG were analyzed by enzymatic assay and measured using a Hitachi 7600 Autoanalyzer (Hitachi Ltd., Tokyo, Japan). ApoB-containing lipoproteins were precipitated with dextran-sulfate magnesium, and HDL-cholesterol concentrations in the supernatants were measured enzymatically. For subjects with serum TG levels <400 mg/dL, LDL-cholesterol concentrations were estimated indirectly using the Friedwald formula: LDL-cholesterol = Total-cholesterol—[HDL-cholesterol + (TG/5)]. For subjects with serum TG levels ≥400 mg/dL, LDL-cholesterol concentrations were measured directly using a Hitachi 7600 Autoanalyzer. Free fatty acid (FFA) was analyzed by enzymatic assay [the acylCoA synthetase-acylCoA oxidase (ACS-ACOD) method] with a Hitachi 7600 Autoanalyzer.

Fasting glucose levels were analyzed by the hexokinase method using a Hitachi 7600 Autoanalyzer. Insulin levels were measured by a radioimmunoassay using commercial kits from the Immuno Nucleo Corporation (Stillwater, MN, USA). Insulin resistance (IR) was calculated by the homeostatic model assessment (HOMA) method using the following equation: HOMA–IR = [Fasting insulin (μIU/mL) × Fasting glucose (mmol/L)] / 22.5. All subjects underwent an oral glucose tolerance test (OGTT) by ingesting a 75-g glucose solution after a 12-hour overnight fast. Venous specimens were collected before glucose loading, at loading, and 30, 60, and 120 min after loading to determine the serum glucose levels and responses. Insulin and FFA levels and responses were also determined.

The concentration of serum high-sensitivity C-reactive protein (hs-CRP) was measured with an Express Plus autoanalyzer (Chiron Diagnostics Co., Walpole, MA, USA) using a commercially available, high-sensitivity CRP-Latex(II) X2 kit (Seiken Laboratories Ltd., Tokyo, Japan). LDL particles were isolated by sequential flotation ultracentrifugation, and the particle size distribution (1.019–1.063 g/mL) was examined using a pore-gradient lipoprotein system (CBS Scientific Company, San Diego, CA, USA) on commercially available non-denaturing gels containing a linear 2–16% acrylamide gradient (CBS Scientific Company). Latex bead (30 nm)-conjugated thyroglobulin (17 nm), ferritin (12.2 nm), and catalase (10.4 nm) standards were used to estimate the relative band migration rates. Gels were scanned using a GS-800 Calibrated Imaging Densitometer (Bio-Rad Laboratories, Hercules, CA, USA). Plasma oxidized (ox)-LDL was measured using an enzyme immunoassay (Mercodia AB, Uppsala, Sweden), and the resulting color reaction was determined at 450 nm on a Wallac Victor^2^ multilabel counter (Perkin-Elmer Life Sciences, Boston, MA, USA). Plasma malondialdehyde (MDA) was measured from thiobarbituric acid-reactive substances (TBARS) using the TBARS Assay Kit (ZeptoMetrix Co., Buffalo, NY, USA).

### Global metabolic profiling

#### Plasma extract sample preparation

Prior to analysis, 800 μL of 80% acetonitrile (J.T.Baker, HPLC grade) was added to 100 μL of plasma, placed at 4°C for 10 min, mixed by vortexing gently, and centrifuged at 10,000 rpm for 5 min at 4°C. Then, 820 μL of supernatant was dried with N_2_ (l), dissolved in 100 μL of cold 10% methanol (J.T.Baker, HPLC grade), mixed by vortexing for 1 min, and centrifuged at 10,000 rpm for 5 min at 4°C. Finally, 85 μL of supernatant was transferred into a vial.

#### Ultra-performance liquid chromatography (UPLC) and linear-trap quadrupole (LTQ) Orbitrap XL mass spectrometry

Plasma extract samples (4 μL) were injected into an Acquity UPLC-BEH-C18 column (2.1 × 50 mm, 1.7 μm, Waters, Milford, MA), which was coupled in-line with a UPLC-LTQ-Orbitrap XL (Thermo Fisher Scientific, Waltham, MA). The injected samples were equilibrated with water (Brudick & Jackson, HPLC grade) containing 0.1% formic acid (Sigma Aldrich, 99%) (solvent A) and then eluted through an acetonitrile (Tedia, 99.9%) gradient containing 0.1% formic acid (solvent B) at a flow rate of 0.35 mL/min for 20 min. The gradient was 0 min, 1% B; 1 min, 1% B; 7 min, 20% B; 15 min, 90% B; 16 min, 90% B; 16.2 min, 1% B; and 20 min, 1% B. Metabolites were separated by UPLC (Thermo Fisher Scientific, Waltham, MA) and then assigned by the LTQ-Orbitrap-XL (Thermo Fisher Scientific, Waltham, MA). The mass spectrometer was operated in ESI-positive mode, in full scan mode, and with a Fourier transform mass spectrometer (FTMS). The resolution was 30,000, and the spray voltage was 5 kV. The flow rate nitrogen sheath gas and auxiliary gas were 50 and 5 (arbitrary units). The capillary voltage (V), tube-lens voltage (V), and capillary temperature (°C) were kept constant at 35 V, 80 V, and 370°C, respectively. The Orbitrap data were collected in the range of m/z 50–1,000. The samples were randomized before analysis, and a mixture of four standard compounds (acetaminophen, sulfadimethoxine, terfenadine, and reserpine; Sigma Aldrich) was included every tenth sample as a quality control. A blank (70% methanol; J.T.Baker, HPLC grade) was also included every tenth sample for column cleaning. The MS/MS spectra of metabolites were obtained by a collision-energy ramp from 55–65 eV.

#### Data processing and identification of metabolites

All MS data, including the retention times, m/z, and ion intensities, were extracted by SIEVE software (Thermo Fisher Scientific, Waltham, MA) and incorporated into the instrument, and the resulting MS data were assembled into a matrix. The SIEVE parameters were set as follows: m/z range 50–1,000; m/z width 0.02; retention time width 2.5; and m/z tolerance 0.005. Metabolites were searched within the following databases: ChemSpider (www.chemspider.com), Human Metabolome (www.hmdb.ca), Lipid MAPS (www.lipidmaps.org), KEGG (www.genome.jp/kegg), and MassBank (www.massbank.jp). To confirm the putative metabolites, MS/MS was performed. The MS/MS spectra were exported from the Xcalibur 2.1 software (Thermo Fisher Scientific, Waltham, MA) to the MS frontier software (Thermo Fisher Scientific, Waltham, MA) and then compared with references in the MS frontier software database or Human Metabolome, Lipid MAPS, MassBank MS/MS spectra databases.

### Statistical analyses

Statistical analyses were performed using SPSS v. 21.0 (IBM SPSS Statistics 21, Chicago, IL, USA). Skewed variables were logarithmically transformed for statistical analyses. For descriptive purposes, the mean values are presented using the untransformed values. The results are expressed as the mean ± standard error (SE). A two-tailed *P*-value of <0.05 was considered statistically significant. Differences in the clinical variables between the NTG and HTG groups were tested using Student’s independent *t*-tests. General linear model tests were applied to the clinical variables to adjust for potential confounding factors, including age, BMI, WHR, smoking, drinking, systolic and diastolic BP, total- and LDL-cholesterol, glucose, and insulin. Pearson’s correlation coefficients were computed to examine the relationships among the variables. Multiple regression analyses were performed to identify the major plasma metabolites associated with the fasting TG level. False discovery rate-corrected *q*-values were computed using the R package ‘fdrtool’. Heat maps were created to visualize and evaluate the correlations among the metabolites and clinical characteristics in the two groups. Red represents a positive correlation, and blue represents a negative correlation among the variables.

Multivariate statistical analysis was performed using SIMCA-P+ software version 12.0 (Umetrics, Umeå, Sweden). A partial least-squares discriminant analysis (PLS-DA) was used as a classification method to model the discrimination between groups by visualizing the score scatter plot or *S*-plot using the first and second PLS components. PLS-DA consists of a classical PLS regression in which the dependent variable y is categorical and represents a sample’s class membership. The large number of peaks in these spectra, all of which are potential biomarkers, create modeling and validation challenges. The PLS-DA score scatter plot is a summary of the relationships among the samples, and it involves the projection of the data onto two dimensions. Each data point in the score scatter plot represents a spectrum. In this plot, samples that are close are similar, and samples that are distant are dissimilar. An *S*-plot was used to visualize both the covariance and the correlation from the PLS-DA model in a score scatter plot. In the *S*-plot, the p[1]-axis describes the magnitude of each variable in X, and the p(corr)[1]-axis represents the reliability of each variable in X. High reliability indicates a strong effect and low uncertainty indicates a putative biomarker, and their variable importance in the projection (VIP) values are high.

Two types of validation were conducted to validate the model. First, a sevenfold validation was applied to the PLS-DA model. The data were divided into 7 parts, and each 1/7^th^ was removed in turn. A model was built on the 6/7^th^ remaining data, and the excluded data were predicted from the new model. This process was repeated with each 1/7^th^ of the data until all of the data were predicted. The predicted data were then compared with the original data, and the sum of the squared errors was calculated for the whole dataset. This result was then called the Predicted Residual Sum of Squares (PRESS). Better predictability of the model has a lower PRESS value. For convenience, we converted PRESS into *Q* ^*2*^ to resemble the *R* ^*2*^ scale. PRESS was divided by the initial sum of squares and subtracted from 1. Good predictions have a low PRESS and high *Q* ^*2*^. The goodness of the fit was quantified by *R* ^*2*^, whereas the predictive ability was quantified by *Q* ^*2*^. Generally, R^2^ describes how well the data in the training set are mathematically reproduced and varies between 0 and 1 (a value of 1 indicates a model with a perfect fit). Models with *Q* ^*2*^ *Y* ≥0.5 are considered to have good predictive capabilities. Second, the reliabilities of the model were further rigorously validated by a permutation test (n = 200). This test evaluated the significance of the estimated predicted power of the models by comparing the *R* ^*2*^ and *Q* ^*2*^ values of the original model with the values in the re-ordered model, which was created anew whenever the Y data were permutated at random. Models with a *R* ^*2*^ intercept less than 0.3–0.4 and a *Q* ^*2*^ intercept less than 0.05 were valid models [7].

## Results

### Clinical characteristics and nutrient intake of NTG and HTG subjects

The mean fasting TG levels in the HTG group were significantly higher than those in the NTG group (Table 1). The mean fasting TG level for the first measurement was 103 mg/dL in the NTG group compared with 220 mg/dL in the HTG group. The HTG subjects exhibited a slightly but significantly higher systolic BP. After adjusting for age, BMI, WHR, smoking, drinking, BP, total- and LDL-cholesterol, glucose, and insulin, the HTG group exhibited a higher HOMA-IR index (*P*<0.001), TG level (*P*<0.001), fasting FFA (*P* = 0.034), the FFA response area during OGTT (*P =* 0.006), and ox-LDL (*P*<0.001), as well as a reduced HDL-cholesterol level (*P*<0.001) and a smaller LDL particle size (*P*<0.001), than those of the NTG group (Table 1). There were no differences in the estimated total calorie intake between the two groups (NTG: 2,059 ± 20 kcal/d; HTG: 2,070 ± 19 kcal/d). No significant differences were found with respect to the proportion of calorie intake from macronutrients between the two groups (data not shown). For example, the polyunsaturated/monounsaturated/saturated (P/M/S) fat intake ratio between the NTG (1:0.54:0.37) and HTG (1:0.58:0.38) groups was not significantly different. In addition, there were no significant differences in the ratio of total energy intake to total expenditure between the two groups (data not shown).

**Table 1:** Clinical characteristics of the study subjects.

	Normotriglyceridemia (n = 111)	Hypertriglyceridemia (n = 111)	***P***	***P***_*1*_
**Age (years)**	53.5±0.80	52.3±0.79	0.294	-
**Male/female (%)**	16.2 / 83.8	16.2 / 83.8	1.000	-
**Body mass index (kg/m** ^**2**^)	24.0±0.24	24.7±0.24	0.075	-
**Waist-to-hip ratio**	0.91±0.01	0.91±0.01	0.982	-
**Cigarette smoker, n (%)**	7 (7.4)	11 (10.2)	0.481	-
**Alcohol drinker, n (%)**	48 (43.2)	59 (53.2)	0.140	-
**Systolic BP (mmHg)**	117.2±1.28	121.1±1.25	0.029	-
**Diastolic BP (mmHg)**	77.8±0.83	80.0±0.90	0.068	-
**Total-cholesterol (mg/dL)** ^***∮***^	204.5±3.10	212.0±3.49	0.125	-
**LDL-cholesterol (mg/dL)** ^***∮***^	127.3±2.77	120.5±3.47	0.052	-
**Glucose (mg/dL)** ^***∮***^	85.2±0.79	88.0±1.06	0.048	-
**Insulin (μIU/mL)** ^***∮***^	8.97±0.29	10.1±0.43	0.111	-
**HOMA-IR** ^***∮***^	1.90±0.07	2.21±0.10	0.042	<0.001
**Triglyceride (mg/dL)** ^***∮***^	106.6±2.51	222.6±7.56	<0.001	<0.001
**HDL-cholesterol (mg/dL)** ^***∮***^	56.0±1.00	48.4±0.94	<0.001	<0.001
**Free fatty acid (μEq/L)** ^***∮***^	575.3±20.3	598.9±16.8	0.164	0.034
**Glucose response area (mg/dL×h)** ^***∮***^	272.0±5.30	282.5±6.18	0.235	0.200
**Insulin response area (μIU/dL×h)** ^***∮***^	125.7±5.86	138.0±8.52	0.665	0.818
**Free fatty acid response area (μEq/L×h)** ^***∮***^	485.0±16.9	531.7±16.5	0.024	0.006
^**1**^ **hs-CRP (mg/dL)** ^***∮***^	1.25±0.27	1.22±0.10	0.205	0.834
**LDL particle size (nm)** ^***∮***^	23.7±0.06	23.2±0.07	<0.001	<0.001
**Oxidized LDL (U/L)** ^***∮***^	43.5±1.08	49.1±1.44	0.002	<0.001
**Malondialdehyde (nmol/mL)** ^***∮***^	7.27±0.11	7.91±0.17	0.004	0.985

Data are reported as the mean±SE.

^∮^Data were log-transformed prior to analysis.

*P*-values were derived from an independent t-test.

*P*_*1*_-values were derived from an independent t-test after adjusting for age, BMI, waist-to-hip ratio, smoking, drinking, systolic and diastolic blood pressure, total- and LDL-cholesterol, glucose, and insulin.

^1^hs-CRP = high sensitivity C-reactive protein.

### Plasma metabolic profiling based on UPLC-LTQ-Orbitrap MS

#### Global metabolic profiling pattern analyses

**Fig 1:**
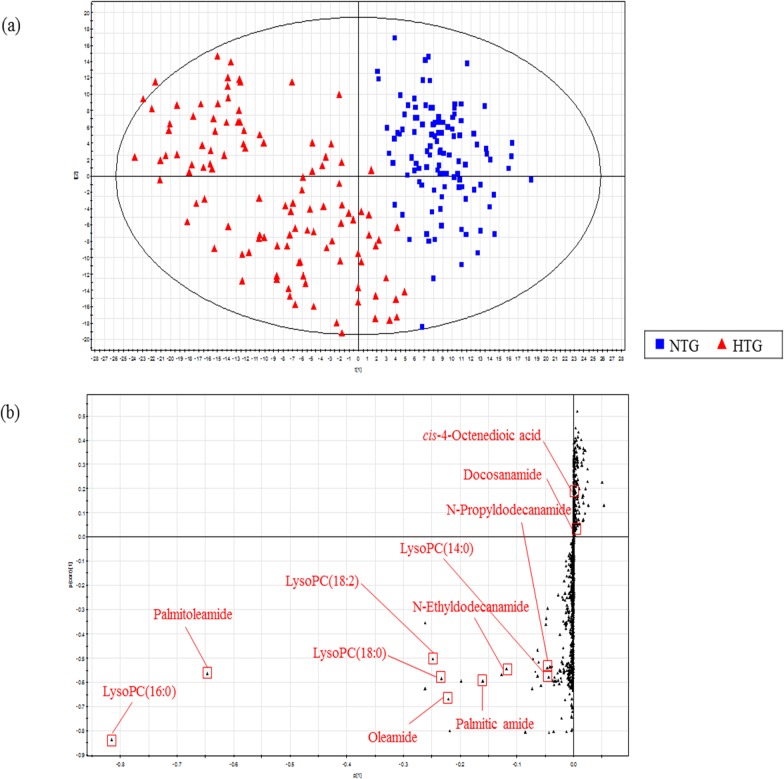
Identification of plasma metabolites in the NTG and HTG groups. (a) Score scatter plot from the PLS-DA model for the NTG group (n = 111) and HTG group (n = 111). (b) *S*-plot for the covariance [p] and reliability correlation [p(corr)] from the PLS-DA model.

The MS data of the plasma metabolites obtained from the NTG and HTG subjects were applied to a PLS-DA score scatter plot (Fig 1A). The two-component PLS-DA score scatter plot of the plasma metabolites exhibited distinct clustering and clear separation for each group. Both groups could be clearly differentiated by the primary component t(1) or the secondary component t(2) based on the model with *R* ^2^ *X*(cum) and *R* ^2^ *Y*(cum) values of 0.119 and 0.806, respectively, which indicated a good fit of the data. The *Q* ^2^ *Y*(cum) value of 0.721 provided an estimate of the predictive ability of the model. The PLS-DA model was validated using a permutation test, which indicated an *R* ^2^ *Y* intercept of 0.339 and a *Q* ^2^ *Y* intercept of -0.17. To identify the metabolites that contributed to the ability to differentiate between the NTG and HTG groups, an *S*-plot of p(1) and p(corr)(1) was generated using centroid scaling (Fig 1B). The centroid is particularly useful when all variables are of the same type and their numerical size and intrinsic variation carry information (e.g., spectral data) [7]. If a large number of variables have low variation, they cannot be interpreted broadly. Using centroids and absolute variation, biomarkers can be determined precisely. The *S*-plot revealed that the metabolites with higher or lower p(corr) values served as the more relevant metabolites for discriminating between the two groups.

#### Identification of plasma metabolites

The metabolites (variables) that differentiate the NTG and HTG groups were selected according to their VIP parameter. VIP values >1.0 indicate variables that are highly associated with differences between the sample groups. Among 1,782 plasma metabolites, 89 metabolites were selected based on VIP values >1.0. Of these metabolites, 11 identified, and 78 were unknown (Table 2). Among the 11 identified plasma metabolites, the normalized peak intensities of 4 amides, including N-ethyldodecanamide (*q* = 2.9E-05), N-propyldodecanamide (*q* = 3.5E-05), palmitoleamide (*q* = 2.9E-06), and palmitic amide (*q* = 0.019), as well as 2 lysoPCs containing C14:0 (*q* = 0.001) and C16:0 (*q* = 1.8E-05), were significantly elevated in the HTG group compared with the NTG group. In addition, the HTG group exhibited reduced levels of *cis*-4-octenedioic acid (*q*<1.0E-9) and docosanamide (*q* = 0.002).

**Table 2:** Identification of plasma metabolites in normotriglyceridemia and hypertriglyceridemia.

Identity	Formula	Exact mass (M+H)	Normalized peak intensities		***q***-value	VIP
			Normotriglyceridemia (n = 111)	Hypertriglyceridemia (n = 111)		
***Cis*-4-octenedioic acid**	C_8_H_12_O_4_	173.0814	62086±2585	26923±1596	<1.0x10^-9^	1.6553
**N-ethyldodecanamide**	C_14_H_29_NO	228.2327	223860±15562	367283±28465	2.9x10^-5^	4.4782
**N-propyldodecanamide**	C_15_H_31_NO	242.2484	81530±6027	137912±11355	3.5x10^-5^	1.7589
**Palmitoleamide**	C_16_H_31_NO	254.2484	379346±25298	621220±41393	2.9x10^-6^	18.5158
**Palmitic amide**	C_16_H_33_NO	256.2640	391859±25948	486361±32370	0.019	3.5773
**Oleamide**	C_18_H_35_NO	282.2797	2474611±120737	2640786±130255	0.148	7.2966
**Docosanamide**	C_22_H_45_NO	340.3579	235681±16915	174776±9009	0.002	2.9816
**LysoPC (14:0)**	C_22_H_46_NO_7_P	468.3090	211506±8941	251513±7201	0.001	1.1946
**LysoPC (16:0)**	C_24_H_50_NO_7_P	496.3403	6411140±95638	7088030±113274	1.8x10^-5^	21.1369
**LysoPC (18:2)**	C_26_H_50_NO_7_P	520.3403	2460128±54505	2391276±54027	0.164	9.4772
**LysoPC (18:0)**	C_26_H_54_NO_7_P	524.3716	2081610±39671	2151665±47108	0.126	6.3785

Data are reported as the mean±SE.

The *q*-value was derived from an independent t-test.

### Correlations among the fasting TG level, clinical and biochemical parameters, and major plasma metabolites

**Fig 2:**
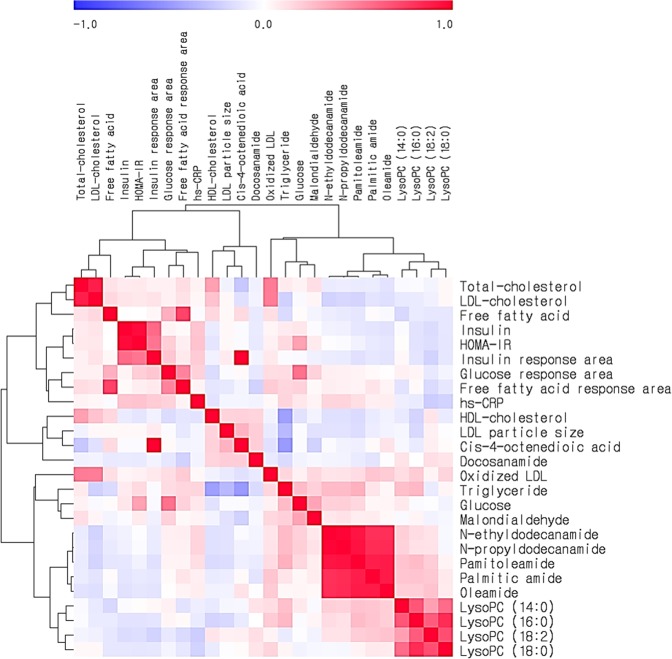
Correlation matrix of the fasting triglyceride levels, clinical and biochemical parameters, and major plasma metabolites. The supervised hierarchical clustering plot shows that the 11 most important metabolites stratify the samples according to 15 clinical characteristics. The correlations were obtained by deriving Spearman correlation coefficients. *Red* represents a positive correlation, and *blue* represents a negative correlation.

The correlation matrix among the fasting TG level, clinical and biochemical parameters, and levels of major plasma metabolites were computed for each subject (n = 222) (Fig 2). In all subjects, the fasting TG level correlated positively with BMI, BP, glucose, the glucose response area, HOMA-IR index, the FFA response area, hs-CRP, MDA, and ox-LDL and correlated negatively with HDL-cholesterol and LDL particle size. The fasting TG level correlated positively0020with N-ethyldodecanamide, N-propyldodecanamide, palmitoleamide, and lysoPCs containing 14:0 and 16:0 and correlated negatively with *cis*-4-octenedioic acid and docosanamide. Additionally, ox-LDL correlated positively with lysoPCs containing 14:0, 16:0, and 18:0 and several primary fatty acid amides, including palmitoleamide, palmitic amide, and oleamide, and correlated negatively with *cis*-4-octenedioic acid. Based on these results, we performed a multiple regression to identify independent predictors of the fasting TG level. BMI, systolic BP, fasting glucose, the glucose response area, HOMA-IR index, the FFA response area, HDL-cholesterol, N-ethyldodecanamide, N-propyldodecanamide, palmitoleamide, lysoPC (14:0), lysoPC (16:0), *cis*-4-octenedioic acid, and docosanamide were tested. LysoPC (16:0) (standardized *β* = 0.159, *P* = 0.025), *cis*-4-octenedioic acid (standardized *β* = -0.411, *P*<0.001), glucose (standardized *β* = 0.179, *P* = 0.006), the FFA response area (standardized *β* = 0.142, *P* = 0.022), and HDL-cholesterol (standardized *β* = -0.296, *P*<0.001) emerged as independent predictors of the fasting TG level.

## Discussion

This study suggested that borderline-to-moderate HTG is associated with alterations in lipid metabolism in non-diabetic and non-obese subjects. A number of metabolites exhibited increased concentrations in HTG subjects compared with NTG controls, including lysoPCs containing C14:0 and C16:0, N-ethyldodecanamide, N-propyldodecanamide, palmitoleamide, and palmitic amide. In addition, several other metabolites, including *cis*-4-octenedioic acid and docosanamide, exhibited decreased levels in HTG subjects compared with NTG controls.

LysoPC constitutes only 1–5% of the total phosphatidylcholine (PC) content of non-ox-LDL; however, as much as 40–50% of the PC contained within the LDL molecule is converted to lysoPC during LDL oxidation [8]. A saturated fatty acid or a monounsaturated fatty acid predominates in the *sn*-1 position of the phospholipid [9]. Although there was no significant difference in LDL-cholesterol between the HTG and NTG groups, higher ox-LDL levels were observed in the HTG group and were positively correlated with the fasting TG levels, lysoPCs containing C14:0, C16:0, and C18:0, and primary fatty acid amides such as palmitoleamide, palmitic amide, and oleamide. This result supports the findings of a recent study, in which a strong positive correlation between changes in lysoPCs and changes in ox-LDL without changes in LDL-cholesterol was observed [10]. In addition, the current study identified lysoPC 16:0 (VIP value of 21.1369) as the best predictor among the plasma lysoPC metabolites for discriminating between the HTG and NTG groups. VIP values estimate the importance of each variable in the projection used in a PLS model and are often used for variable selection. In addition, the VIP value represents the ability of each predictor to fit the PLS model for both predictors and responses. A variable with a VIP value close to or greater than 1 can be considered important in a given model. Variables with VIP values significantly less than 1 are less important and might be good candidates for exclusion from the model because they only minimally contribute to the prediction [11, 12]. Several studies have reported that lysoPC concentrations in the blood are elevated in a number of pathophysiological conditions, including atherosclerosis [8, 13], hypertension [14], obesity [15, 16], and hyperlipidemia [10, 17]. In obese and lean mice, groups could clearly be discriminated based on the PLS-DA score plot, and lysoPC (16:0) showed the highest VIP value (11.97) [15]. In another study, obese mice that were maintained on a high-fat diet for 14 weeks showed a significant increase in TG levels and saturated lysoPCs [16].

The current study found positive correlations between lysoPCs, fasting TG levels, and several amides, including palmitoleamide. Palmitoleamide (VIP value of 18.5158), which is the primary amide of palmitoleic acid, was identified as the best predictor among the plasma amide metabolites for discriminating between the NTG and HTG groups. The HTG group exhibited an increase in palmitoleamide and palmitic amide and a decrease in docosanamide, another primary fatty acid amide. The mechanism by which docosanamide is produced and degraded in biological systems is unknown (www.hmdb.ca). The major route for primary fatty acid amide degradation *in vivo* is the hydrolysis of fatty acids and ammonia, a reaction that is catalyzed by fatty acid amide hydrolase [18]. One proposed route for primary fatty acid amide biosynthesis is the ammonolysis of fatty acyl-CoA thioesters [19]. A second proposed route involves the oxidative cleavage of N-fatty acylglycines [20, 21]. A recent study has suggested that the endogenous levels of primary fatty acid amides reflect a steady-state balance between production, degradation, and secretion [22]. Furthermore, the endogenous levels of primary fatty acid amides are likely influenced by their respective fatty acids [22]. Fatty acid amides, which are a large family of structurally diverse molecules in humans, are bioactive and represent a novel class of signaling lipids. In the current study, the HTG group exhibited a significant increase in the fasting FFA and FFA response area, which were positively and independently correlated with the fasting TG levels. These results suggest that alterations in the plasma fatty acid amides may reflect changes in their respective fatty acids in the HTG group. Lipoprotein-associated phospholipase A_2_ (Lp-PLA_2_), a member of the phospholipase A_2_ family of enzymes, hydrolyzed oxidized phospholipids to produce lysoPC and oxidized nonesterified free fatty acids [3]. In present study, oxidized LDL, FFA, and lysoPC (16:0) were significantly increased in the HTG group. Additionally, oxidized LDL was positively correlated with lysoPC (16:0) and fatty acid amides, including palmitoleamide and palmitic amide. Taken together, these pathways and correlations based on experimental data could partly explain the relationship between lysoPC (16:0) and palmitoleamide, supporting the conclusion that these two metabolites were the best plasma metabolite predictors for discriminating between the NTG and HTG groups. Although many of these molecules remain poorly defined, several reports have emphasized the importance of fatty acid amides for the early detection of chronic diseases [23–25].

As expected, the HTG group exhibited a smaller LDL particle size and lower concentrations of HDL-cholesterol compared with those of the NTG group. This phenotype, which is called atherogenic dyslipidemia, is observed predominantly in IR [26]. In this study, the HTG group also exhibited a higher HOMA-IR index; however, the HOMA-IR index was not an independent predictor of the fasting TG levels. In contrast, plasma *cis*-4-octenedioic acid was negatively and independently associated with the fasting TG levels. The precursors of octenedioic acid are decenedioic acid and dodecenedioic acid [27]. The metabolic origin of *cis*-4-octenedioic acid is linoleic acid through the stepwise omega- and beta-oxidations of the metabolic intermediate *cis*-4-decenoic acid [28]. Rats treated with hypoglycin, an inhibitor of fatty acid beta-oxidation, exhibited massive dicarboxylic aciduria, including *cis*-4-octenedioic acid, as a consequence of inhibited fatty acid oxidation [29]. A disproportionate increase in decenedioic, dodecenedioic, and octenedioic acids was also found in the plasma or urine of patients with a medium-chain acyl-CoA dehydrogenase deficiency [27, 30]. In this study, the fat percent energy intake and the ratio of polyunsaturated, monounsaturated, and saturated fats were not significantly different between the NTG and HTG groups. In addition, a significant decrease in *cis*-4-octenedioic acid was found in the HTG group. Taken together, these results suggest that individuals with borderline-to-moderate HTG may have an increase in the oxidation of linoleic acid.

We believe that the explanatory power of HOMA-IR for the fasting TG levels in the regression model was weaker than that of other variables that were indicated as independent predictors of the fasting TG levels, such as lysoPC (16:0), *cis*-4-octenedioic acid, glucose, the FFA response area, and HDL, suggesting that the fasting TG levels are explained more by these variables than by HOMA-IR. In addition to this result, the HTG group in the present study showed metabolic differences in lysoPCs and fatty acid amides but not in amino acids compared to the NTG group, while metabolic changes in amino acids, particularly branched-chain amino acids (BCAAs), were predominant in studies related to IR [31], impaired glucose tolerance [32], and T2D [33]. A broad range of amino acids, including leucine, isoleucine, valine, phenylalanine, tyrosine, alanine, tryptophan, and homocitrulline, have been shown to be substantially increased in T2D patients and in subjects with obesity or impaired glucose tolerance [32]. Additionally, higher plasma levels of BCAAs and aromatic acids were associated with an increased risk of T2D in the Framingham Offspring Study [33]. Another study found that increased diacyl-phosphatidylcholines and reduced acyl-alkyl- and lysoPCs together with sphingomyelins were associated with diabetes in a European population [34]. Using a lipidomics-oriented panel with 140 metabolites from a longitudinal population-based study that covered a time span of 7 years, the levels of the three metabolites glycine, lysoPC (18:2), and acetylcarnitine were found to be altered in individuals with impaired glucose tolerance [35]. Changes in blood concentrations of select essential amino acids and their derivatives, in particular BCAA, sulfur amino acids, tyrosine, and phenylalanine, are apparent with obesity and insulin resistance, often before the onset of clinically diagnosed T2D [31]. This result suggests that the metabolic differences between the two groups in our study were driven by TG levels rather than IR, even though HOMA-IR was higher in the HTG group in the present study, and the elevated TG levels were associated with increased insulin levels and IR in other studies [36, 37].

This study has several limitations. The study design was a cross-sectional observational study in which we evaluated associations rather than prospective predictions. Therefore, the causal relationship between the identified metabolites and the exact mechanisms underlying the changes in these metabolites in non-obese and non-diabetic individuals with borderline-to-moderate HTG remain unclear. In addition, although a large number of markers were detected using UPLC-LTQ-Orbitrap MS, most of these markers remain unknown. Although large databases for GC-MS-based techniques exist, databases of endogenous biomolecules for LC-MS-based techniques remain to be constructed [38]. Despite this limitation, our study identified a cluster of borderline-to-moderate HTG-associated changes in plasma metabolites using a UPLC-LTQ-Orbitrap MS-based metabolomics strategy and multivariate data analysis.

## Conclusions

The differences observed between the metabolomic profiles of the NTG and borderline-to-moderate HTG subjects may provide novel insights into the metabolic alterations during the early stages of HTG as a precursor to severe HTG, T2D, and atherosclerosis. The results of this study may therefore facilitate the identification of new intervention targets.

## Supporting Information

S1 Table Identification of unknown plasma metabolites in normotriglyceridemia and hypertriglyceridemia. (XLSX)
